# Combined intake of blueberry juice and probiotics ameliorate mitochondrial dysfunction by activating SIRT1 in alcoholic fatty liver disease

**DOI:** 10.1186/s12986-021-00554-3

**Published:** 2021-05-10

**Authors:** Houmin Fan, Yanyan Shen, Ya Ren, Qiuju Mou, Tao Lin, Lili Zhu, Tingting Ren

**Affiliations:** 1 Guizhou Medical University, Guiyang, Guizhou China; 2 Tongren Maternal and Child Health Care Hospital, Tongren, Guizhou China; 3 Department of Blood Transfusion, The Affiliated Baiyun Hospital of Guizhou Medical University, Guiyang, Guizhou China; 4 Department of Clinical Examination, The Affiliated Hospital of Guizhou Medical University, No 28, Guiyi Street, Yunyan District, Guiyang, Guizhou China; 5 Department of Blood Transfusion, The Affiliated Hospital of Guizhou Medical University, Guiyang, Guizhou China

**Keywords:** Alcoholic fatty liver disease, Blueberry, Probiotics, Mitochondrial dysfunction, SIRT1

## Abstract

**Background:**

Mitochondrial dysfunction has been implicated as a significant factor in the liver disease process. Blueberry juice and probiotics (BP) synergistically improve liver function in alcoholic fatty liver disease (AFLD), although the mechanism for this effect was unclear. This study aims to investigate the effect and specific mechanisms of BP on AFLD.

**Methods:**

C57/BL6 mice were randomly divided into seven groups: CG (control), MG (AFLD model), BJ (MG mice treated with blueberry), BJB (MG mice treated with BP), SI (AFLD mice treated with SIRT1 siRNA), BJSI (SI mice treated with blueberry), and BJBSI (SI mice treated with BP). The mice were fed an alcohol liquid diet for 10 days to establish the AFLD model, and subjected to BP and SIRT1 siRNA intervention for 10 days. Liver pathology was performed on day 11, and biochemical and molecular analyses of liver mitochondria were employed on day 12.

**Results:**

BP significantly ameliorated hepatic mitochondrial injury, mitochondrial swelling, and hepatic necrosis in AFLD. BP alleviated hepatic mitochondrial dysfunction by increasing the expression of succinate dehydrogenase and cytochrome c oxidase, increasing respiratory control rate and the ADP/O ratio, and facilitating the synthesis of energy-related molecules. Besides, BP increased the expression of glutathione and superoxide dismutase, and inhibited malondialdehyde expression and reactive oxygen species activity. BP-induced sirtuin 1 (SIRT1), which activates peroxisome proliferator-activated receptor-gamma coactivator-1α, both of which mediate mitochondrial homeostasis. SIRT1 silencing suppressed the BP-induced changes in liver mitochondria, blunting its efficacy.

**Conclusions:**

The ingredients of BP ameliorate hepatocyte mitochondrial dysfunction in AFLD mice.

**Supplementary Information:**

The online version contains supplementary material available at 10.1186/s12986-021-00554-3.

## Introduction

As the primary site of ethanol metabolism, the liver is the most vulnerable to chronic alcohol intake. Ethanol abuse leads to alcoholic liver disease [1]. Alcoholic fatty liver disease (AFLD), characterized by excess triglycerides enhancement in the liver, is the earliest stage of alcoholic liver disease. The pathogenesis of AFLD is complex, involving abnormal lipid metabolism, endotoxins, cytokines, and other factors. AFLD has become a serious health problem worldwide with high incidence and mortality [2, but there are no effective therapies.

The mitochondria maintain energy production and stability and regulate redox signals. Hepatocytes carry abundant mitochondria to provide energy and regulate the liver functions [2, 3]. Oxidative stress, one of the main mechanisms of hepatocellular injury, leads to AFLD. Mitochondrial dysfunction, a primary initiator of oxidative stress, serves a critical role in the pathogenesis of AFLD [4]. Increased levels of reactive oxygen species (ROS) damage mitochondrial DNA (mtDNA) and initiate large-scale mtDNA deletions [5]. Chronic alcohol consumption causes severe mitochondrial damage and gradual mtDNA depletion. Ongoing DNA damage and depletion lead to mitochondrial dysfunction [6], and accelerate ROS generation in a vicious circle. Mitochondrial morphology and function are altered by chronic excessive drinking and are considered a hallmark of patients and experimental animals with AFLD [7]. However, there have been few studies on mitochondrial function in AFLD.

Many natural products have been identified for use in the treatment of human diseases [8]. For example, bitter melon extract induces tumor cell death through autophagy and suppresses the growth of breast tumors in vivo [9], augments natural killer cell-mediated tumor-killing activity [10], and modulates the immune response in head and neck cancer [11]. Hawthorn fruit extract inhibits trimethylamine N-oxide (TMAO)-aggravated atherogenic compound by enhancing antioxidant capacity and impairing inflammation in mice [12]. Blueberry, a functional fruit, is the most beneficial diet due to its unique chemical and biological properties, including trace elements, flavonoids, vitamins, glycosides, and polyphenols [13]. Blueberry supplementation attenuates mitochondrial oxidative stress and liver damage [14, 15] and prevents the mitochondrial dysfunction in the liver [16], which plays an important role in protecting the liver and reversing liver fibrosis [17]. Probiotics can also inhibit the progression of AFLD in animal models and patients [18]. We have begun to explore the effect and mechanism of blueberry and probiotics (BP) in combination in the treatment of AFLD.

The NAD^+^-dependent histone deacetylase sirtuin-1 (SIRT1) mediates mitochondrial protection, stress responses, cell cycle regulation, and inflammation through the deacetylation of lysine residues in histone and non-histone proteins [19]. SIRT1 regulates lipid metabolism and mitochondrial biogenesis [20], which are dysregulated in AFLD [21]. In AFLD mice, hepatic deletion of SIRT1 promotes steatosis and inflammation, aggravating disease [22].We demonstrated that BP reduced hepatocyte apoptosis in AFLD mice via SIRT1 pathways [23]. Resveratrol, a major active ingredients of blueberry, is an agonist of SIRT1 activity [24].

Here, we explored the effect and underlying mechanisms by which BP ameliorates AFLD via SIRT1-mediated mitochondrial regulation. We established an animal model of AFLD disease, treated the animals with blueberry and probiotics, and evaluated changes in the hepatocyte mitochondria. We used SIRT1 silencing to verify the role of SIRT1 in AFLD mice treated with BP and measured SIRT1 and peroxisome proliferator-activated receptor-gamma coactivator-1α (PGC-1α), which are involved in mitochondrial dysfunction, to model the regulatory mechanism of BP in AFLD liver mitochondria.

## Materials and methods

### Acquisition of BP

Blueberries (Gardenblue) were obtained from an orchard in Majiang of Guiyang, China. Blueberry juice was extracted using a well-established method [16]. Briefly, blueberries (1 kg) were thawed at 4 °C for 8 h and then ground with a Braun Global Hand Mixer (MR 300; De 'Longhi Kenwood A. P. A. Ltd., Hong Kong, China). The fruit mixture was then squeezed in a bag stamper at a maximum pressure of 0.9 MPa. The obtained juice was immediately administered to the rats. The main components of the blueberry juice were constant, with 0.98 ± 0.07 mg/mL anthocyanin, consistent with our previous results [23]. Dried probiotic tablets containing *Bifidobacterium infantis, B. animalis, and Lactobacillus acidophilus* were purchased from the China General Microbial Culture Collection Center (Beijing, China). The tablets (containing 5 × 10^6^ CFU/mL per tablet) were powdered, blueberry juice (10 mL) was added, and stored at 4 °C. Live bacteria were detected by microscopic evaluation of 0.01 mL blueberry probiotic mixture at 6 h, 12 h, and 24 h. Probiotic survival rate (%) = LG N1/LG N0 × 100% [N1: the count of live bacteria (CFU/mL), N0: the initial count of live bacteria (CFU/mL)]. Bacterial counts were 5.9 × 10^7^ CFU/mL at 6 h, 6.3 × 10^7^ CFU/mL at 12 h, and 5.2 × 10^8^ CFU/mL CFU/mL at 24 h. BP with 10^8^ CFU/mL probiotics was used for all experiments.

### Establishment of the AFLD model

C57BL/6 J mice (half male/female, 6–8 weeks, 15–18 g) were purchased from the Animal Center of Guizhou Medical College [Approval No.: SCXK (G) (Guizhou) 2012–0001, Guiyang, China]. The animals were fed adaptively to the age of 10 weeks, then divided into control group and the model groups. The control group was received the diet of Lieber DeCarli '82 (F1259SP) diet, prepared as 225 g dry F1259SP powder dissolved in 860 mL double-distilled water (ddH_2_O_2_). This feed with the nutrient control diet (kcal/L) containing protein (151 kcal/L), fat (359 kcal/L), and carbohydrate (490 kcal/L) can be refrigerated at 4 °C and used within 3 days. Prior to F1258SP feeding, the model mice were pretreated via stomach injection of 31.5% ethanol solution (5 g solution/kg body weight). The ethanol solution of 6.6 mL 95% ethanol and 13.4 mL ddH_2_O_2_ was used immediately. The ethanol diet was prepared as 133 g F1258SP dissolved in 700 ml ddH_2_O_2_, which 20.3 g maltodextrin added in a final volume of 949 mL. The ethanol diet can be refrigerated at 4 °C for up to 3 days (151 kcal/L protein, 359 kcal/L fat, 135 kcal/L carbohydrates, 355 kcal/L Ethanol, and maltose). The model group was treated for 10 days, during which two mice were sacrificed for pathological examination. The remaining mice were treated with BP and SIRT siRNA in seven groups of eight mice as follows: CG, control group; MG, AFLD model group; BJ, MG group treated with blueberry; BJB, MG group treated with BP; SI, AFLD mice treated with SIRT1 siRNA; BJSI, SI group treated with blueberry; BJBSI, SI group treated with BP.

All animal protocols were approved by the Animal Care and Ethics Committee of the Affiliated Hospital of Guizhou Medical University and performed per the National Institutes of Health Guidelines for the Protection and Use of Laboratory Animals and Animal Welfare Act guidelines.

### Gene silencing by siRNA

SIRT1 siRNA was used to knock down SIRT1. Starting from the SIRT1 initiation codon (AUG), the 19-base sequence at the 3′ end of the “AA or NA” sequence were used as potential targets for siRNA design. Both strands were designed using this sequence (without AA and NA repeats), with the GC content maintained between 30 and 60%. The selected sequences were searched against the BLAST database (www.ncbi.nlm.nih.gov/BLAST/) to avoid homology. The sequence with the best blocking effect (GCAGGTTGCAGGAATCCAAAG) was packaged in a lentiviral plasmid using vector LV3 (H1/GFP and Puro) and Gemma Technology. The viral supernatant was collected, and the titer was calculated 48 h after transfection. AFLD mice received intra-articular injections of 100 μL lentiviral supernatants (1 × 10^8^ viral particles) twice daily for 10 days.

### Treatment with BP

The BP dose used in mice was performed according to our previous experiments [23, 25]. The blueberry groups were treated with blueberry juice (1.5 mL/100 g body weight) every day for 10 days after confirming the AFLD model and the effect of SIRT1 siRNA. The BP groups received intragastric administration of blueberry juice (1.5 mL/100 g body weight) and probiotics (250 mg/100 mL and 20 mL/100 g body) for 10 days.

### Evaluation of the mitochondrial ultrastructure

After 10 days of treatment with BP and SIRT1 siRNA, all mice were sacrificed, and liver tissues were collected. Liver tissue sections were obtained as described [23]. Sections were stained for 5 min with 1% toluidine blue and sealed with rubber, 40–60 nm sections were stained with uranyl acetate for 30 min and analyzed by transmission electron microscope (JEM1200EX, JEOL, Tokyo, Japan).

### Detection of liver mitochondrial swelling

After 10 days of treatment with BP and SIRT1 siRNA, mitochondria was isolated from the liver tissues as described [23]. The mitochondrial was mixed with 1 mL swelling test solution, which contains 0.25 mol/L sucrose, 5 × 10^−3^ mol/L KH_2_PO_4_, 3 × 10^−3^ mol/L sodium, and 3 × 10^−4^ mol/L CaCl_2_. After incubating for 0 min (A0) and 20 min (A20), absorbance was measured at 520 nm and 25° C. The degree of mitochondria swelling is reported as ΔA (ΔA = A0–A20). A larger ΔA value indicates a more robust mitochondrial buffering of calcium ions and better structural integrity.

### Measurement of liver necrosis

After 10 days of treatment with BP and SIRT1 siRNA, liver tissues were collected, washed with PBS, fixed with 4% paraformaldehyde, embedded in paraffin, cut into 4-μm sections, and stained with hematoxylin and eosin. Five different visual fields were randomly screened from each sample under a 200 × field of view. Areas of liver necrosis were measured with an Olympus Bx 41 (Olympus, Tokyo, Japan). Other liver tissues were stored at − 80 °C.

### Evaluation of the mitochondrial respiratory function

On day 12, liver tissues were removed from the − 80 °C freezer to detect the mitochondrial respiratory function. Mitochondrial respiratory function was analyzed in frozen liver sections as the respiratory control ratio (RCR), represented as the ratio of the respiratory rate at ST3 and ST4. The respiratory rate was detected using the Clark Oxygen Electrode technique in a 3-mL volume at pH 7.35–7.45 with 0.01 mol/L KCl, 5 mmol/L MgCl_2_, 0.2 mol/L succinic acid, 0. 01 mol/L Tris-HC1, 0.25 mmol/L sucrose, 5 mmol/L KH_2_PO_4_, 0.33 mmol/L ADP, and 1 g/L mitochondrial protein. The mitochondrial RCR and ADP/oxidation ratio (ADP/O) were calculated.

### Determination of ROS

Liver tissues removed from the − 80 °C freezer at day 12 were used for the determination of ROS. In brief, tissues were placed in cold PBS, then lysed with trypsin solution for 20 min at 37 °C. The supernatant was discarded, the tissues were lysed in IV collagenase for 40 min at 37 °C, and then filtered. The cell suspension was centrifuged for three times for 3 min at 800 × g and 4 °C three times. Cells were collected and incubated with the fluorescent probe DCFH-DA for 30 min, then washed three times with PBS. Fluorescein and 2 mL PBS were added, and the samples were observed by a fluorescence microscopy.

### Immunochemistry (IHC)

Liver tissues removed from the − 80 °C freezer at day 12 were used for IHC analysis. Liver tissues were washed 3 times with PBS, immersed in 4% formaldehyde fixation for 3 h. The tissues were dehydrated in 30% sucrose, embedded in an optimal cutting temperature mixture (Sakura Finetek Co., Ltd, Tokyo, Japan), frozen on dry ice, and then cut into 6-μm thick sections. These sections were treated with target retrieval solution (Dako, Tokyo, Japan) for 45 min in a steamer and blocked with serum-free protein (Dako, Denmark) for 30 min. After overnight incubation at 4 °C with primary antibodies against SIRT1 (Abcam #104833; 7: 1000) and PGC-1α (Abcam #54481; 1: 500), the sections were incubated with goat anti-rabbit antibody (BA1081) for 2 h at room temperature. Antibody binding sites were observed by microscopy with an Olympus BX41, and the mean optical density of five random fields was analyzed by MIAS 2000 (Olympus, Tokyo, Japan).

### Western blot

Total protein in liver tissues, removed from the − 80 °C freezer at day 12, was extracted, and concentrations was determined via a BCA assay kit (Pierce Biotech, Inc., Rockford, USA).

Total protein (30 µg) was separated by 10% SDS-PAGE gel and transferred to polyvinylidene fluoride membranes (Millipore, Bedford, MA, USA). After blocking, the membranes were incubated with primary antibodies (AMPK, 1:1000, ab110036, Abcam; p-AMPK, 1:1000, ab194920, Abcam; SIRT1, 1:2000, ab110304, Abcam; PGC–1α, 1:1000, ab54481, Abcam; GAPDH, 1:1000, Abcam) overnight at 4 °C, and then incubated with goat anti-rabbit IgG-HRP secondary antibody (1:20,000, Abcam) for 2 h. The antigen–antibody complexes were detected by enhanced chemiluminescence reagent (Thermo Fisher Scientific, Waltham, MA), and photographed by the Chemi Doc MP system (Bio-Rad, Hercules, USA). IMAGE J software was used to analyze the optical density.

### Reverse transcription-quantitative polymerase chain reaction

Liver tissues removed from the − 80 °C freezer at day 12 were used for PCR analysis. Total RNA was extracted with trizol reagent, and cDNA was reverse-transcribed in an ABI one-step fast thermocycler using SYBR Premix Ex Taq TM (Takara, Tokyo, Japan) and the following cycling conditions: 1 cycle of 95 °C for 10 min, 40 cycles of 95 °C for 5 s, 55 ~ 58 °C for 30 s. Relative mRNA expression of SIRT1 and PGC-1α were normalized to GAPDH by the 2 − ΔΔCT method.

### Statistical analysis

Each experiment was repeated eight times. Data were obtained from the eight replications and expressed as mean ± standard deviation (SD). Statistical analysis was performed using SPSS software. Differences between groups were calculated using Student’s t-test, and the statistical significance was set at P < 0.05.

### Results

#### SIRT1 deficiency abrogates the effect of BP in AFLD hepatocyte ultrastructure

**Fig. 1:**
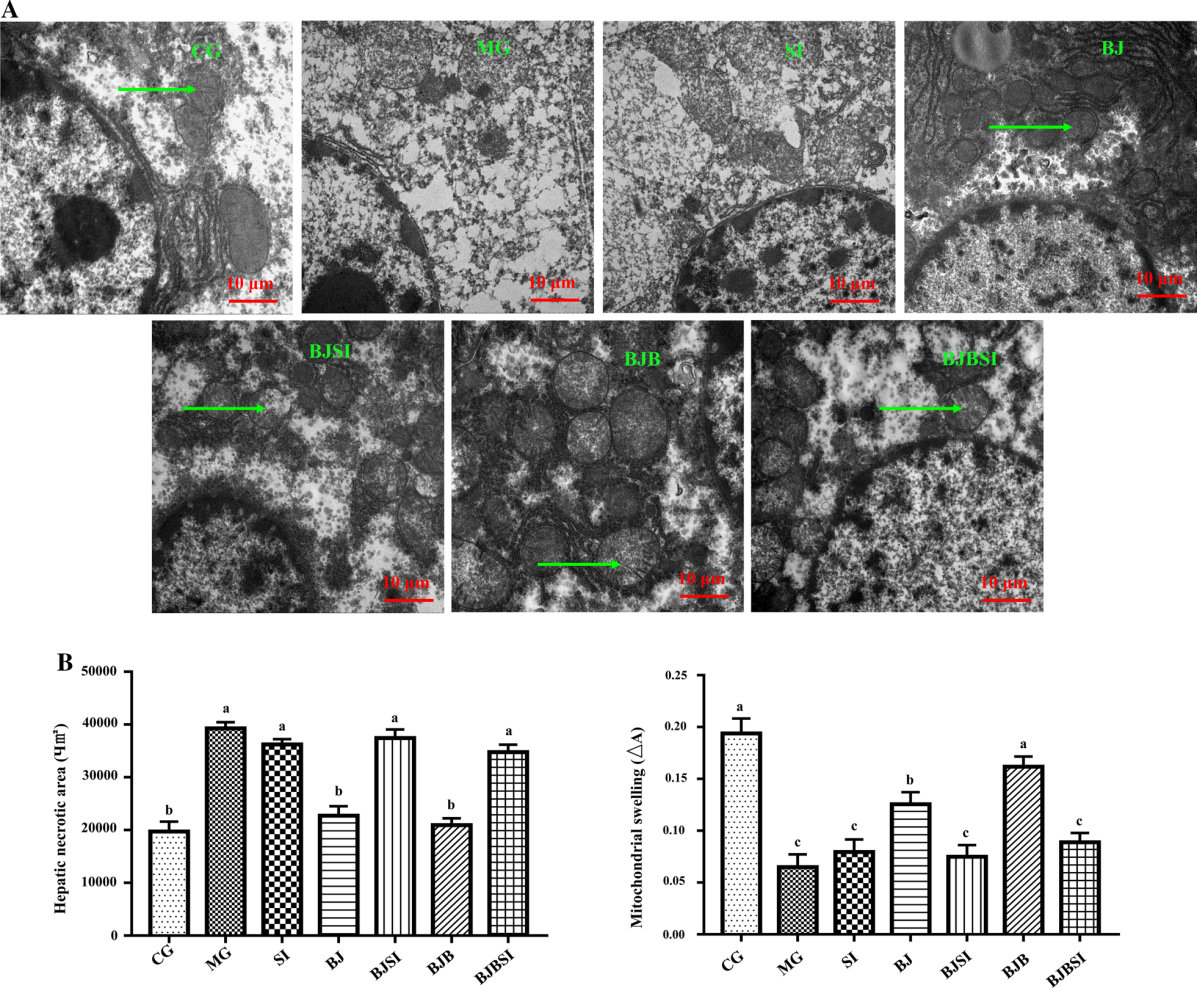
SIRT1 deficiency abrogates the effect of BP in AFLD hepatocyte ultrastructure. **A** Hepatocyte ultrastructure in mice of different groups (Scale bar: 10 μm). The arrow indicates mitochondria; **B** Hepatic necrosis area and mitochondrial swelling. CG, control group; MG, AFLD model group; SI, AFLD mice treated with SIRT1 siRNA; BJ, AFLD mice treated with blueberry; BJSI, AFLD mice treated with blueberry and SIRT1 siRNA; BJB, AFLD mice treated with BP; BJBSI, AFLD mice with BP and SIRT1 siRNA. Different lowercase letters (a, b, c, and d) represent significant differences (*P < 0.05, Student’s t-test, n = 8)

To test the therapeutic effect of BP on the AFLD liver, we observed hepatocyte ultrastructure and mitochondrial integrity in the AFLD mouse liver. The nuclear membrane of normal mouse hepatocytes was intact, the mitochondria showed no noticeable swelling and were clearly visible, with an intact membrane (Fig. 1A). Hepatocytes in AFLD mice were swollen, the nuclear membrane were destroyed, and most damaged organelles appeared damaged, including the mitochondria, which showed a fragmentary and dissolved mitochondrial membrane, with virtually no mitochondrial cristae visible. Treatment with blueberry juice restored the hepatocyte ultrastructure, and BP restored liver mitochondrial integrity (Fig. 1A). Notably, the effect of BP was greater than that of blueberry alone in AFLD mice. Hepatic necrosis was apparent in AFLD mice versus normal controls, but BP reduced the necrotic area and swelling to near-normal levels (Fig. 1B).

To explore the effect of SIRT1 on AFLD, we used siRNA interference. As shown in Fig. 1A, B, SIRT1 interference promoted mitochondrial damage in AFLD mice. To verify that BP efficacy depends on SIRT1, AFLD mice were treated with SIRT1 siRNA and BP, and found that SIRT1deficiency abolished BP protection against AFLD.

#### SIRT1 deficiency limits the effect of BP on mitochondrial function of AFLD mice

**Fig. 2:**
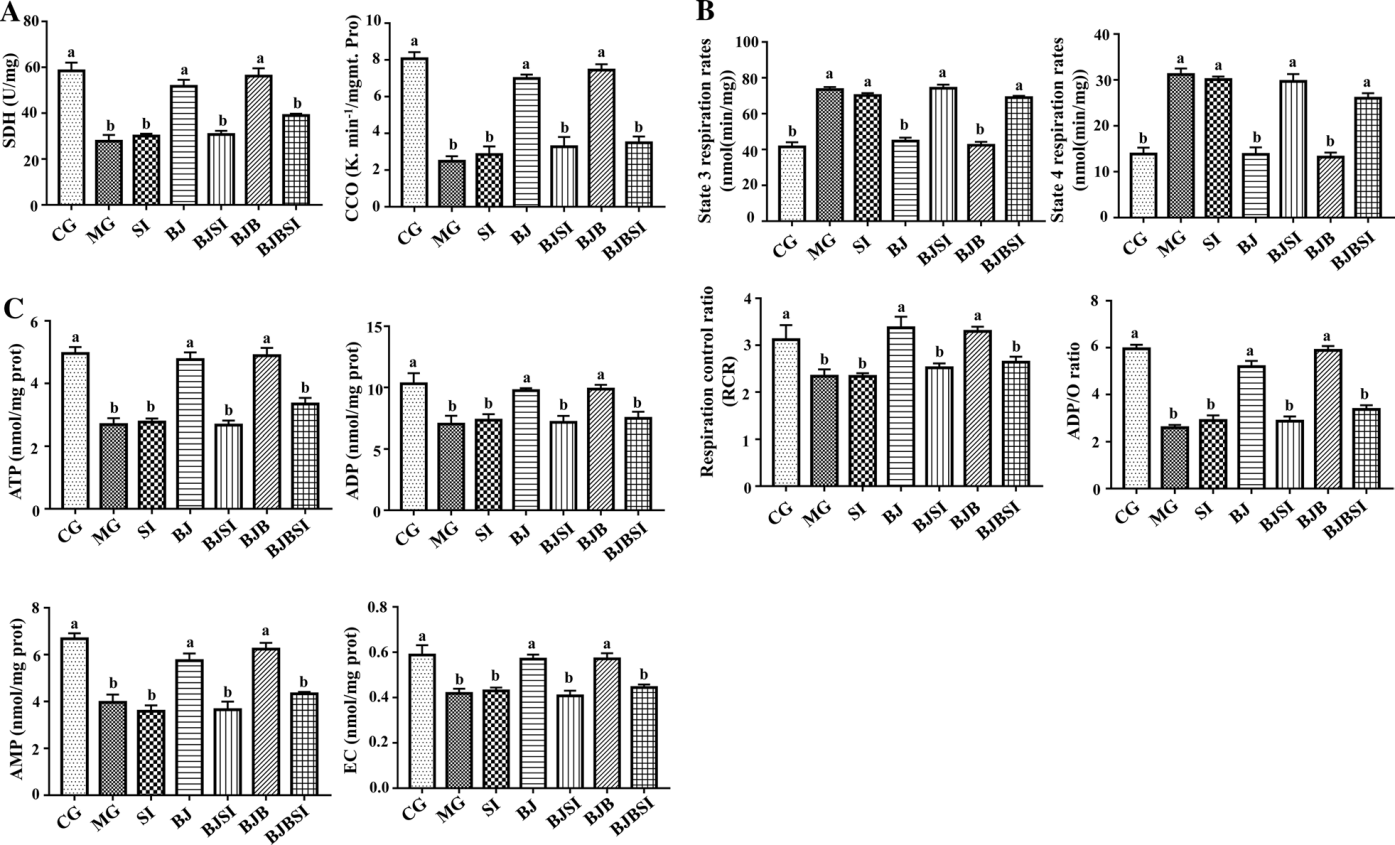
SIRT1 deficiency limits the effect of BP on mitochondrial function of AFLD mice. **A** Functional indexes of hepatic mitochondria were measured using a biochemical marker kit. **B** Hepatic mitochondrial respiratory function is expressed as state 4 and 3 respiration rates, RCR, and the ADP/O ratio. **C** Hepatic mitochondrial synthesis of ATP, ADP, AMP, and EC. Lowercase letters (a, b, c, and d) represent significant differences (*P < 0.05, Student’s t-test, n = 8)

Next, we explored the effect of BP on mitochondrial function in AFLD mice and whether the SIRT1 knockdown impacts this effect. Notably, succinate dehydrogenase (SDH) and cytochrome c oxidase (CCO), indexes of hepatic mitochondrial function, were significantly increased with BP treatment of AFLD mice relative to normal mice, and SIRT1 abrogated this effect (Fig. 2A). Synthesis of hepatic mitochondrial energy metabolism effector molecules (ADP, ATP, AMP, energy charge) was significantly reduced in AFLD, but this was reversed by BP. SIRT1 blockade attenuated mitochondrial energy metabolism (Fig. 2B). We also measured the expression of the energy sensing-related mediators (AMPK, p-AMPK) and found that AMPK level was unchanged, while p-AMPK expression in CG, BJB, and BJ mice was significantly higher than that in the mice with SIRT1 knockdown (Additional file 1: Figure S1).

Furthermore, BP reversed the decline of mitochondrial respiratory function in AFLD mice, restoring RCR and the ADP/O ratio by decreasing the state 4 and state 3 respiration rates. In the presence of SIRT1 siRNA, the respiration rate of mouse state 4 and state 3 was significantly increased and the ratio of RCR and ADP/O was decreased, similar to the AFLD group (Fig. 2C).

#### Knockdown of SIRT1 attenuated the decreasing mitochondrial oxidative stress in AFLD mice with BP treatment

**Fig. 3:**
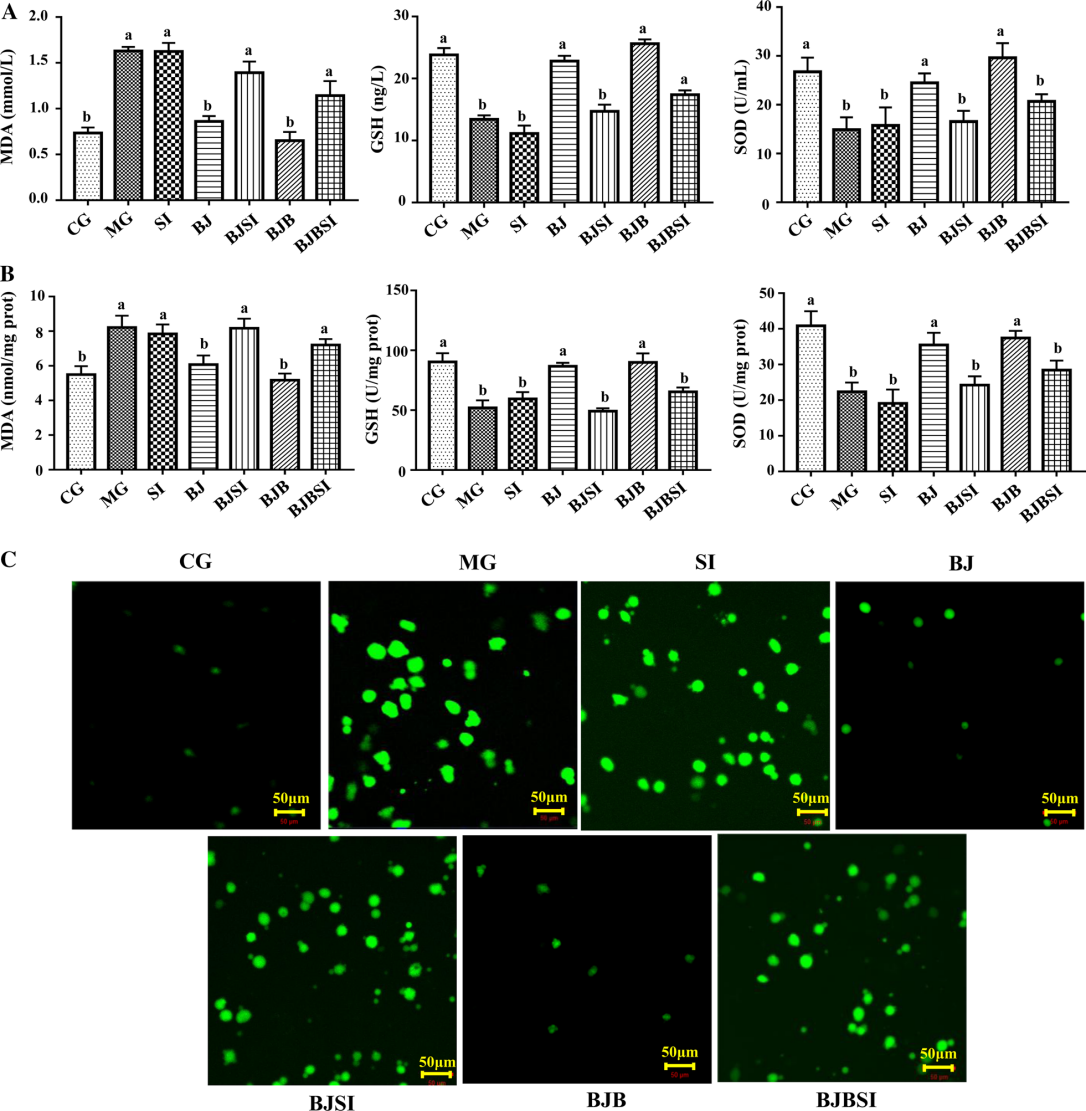
Knockdown of SIRT1 attenuated the decreasing mitochondrial oxidative stress in AFLD mice with BP treatment. **A**, **B** Biomarkers of oxidative stress in liver and hepatic mitochondria. **C** ROS was detected by immunofluorescence assay. Representative photographs of samples from each experimental group (Scale bar: 50 μm). Lowercase letters (a, b, c, and d) represent significant differences (*P < 0.05, Student’s t-test, n = 8)

Oxidative stress, characterized by the characteristic of ROS, increasing malondialdehyde (MDA), and the inhibition of antioxidant enzymes such as superoxide dismutase (SOD) and glutathione (GSH) [26–28], is closely linked to mitochondrial function. Excessive oxidative stress can damage mitochondria, leading to mitochondrial dysfunction[29]. In the liver and hepatic mitochondria of AFLD mice, MDA levels and ROS activity significantly increased, and GSH and SOD expression decreased relative to normal mice (Fig. 3A–C). After treatment with BP, MDA levels were reduced, ROS expression was suppressed, and GSH and SOD levels increased. SIRT1 silencing, however, abolished the effect of BP on mitochondrial oxidative stress in AFLD mice.

#### BP restored the decreased level of SIRT1 in AFLD mice

**Fig. 4:**
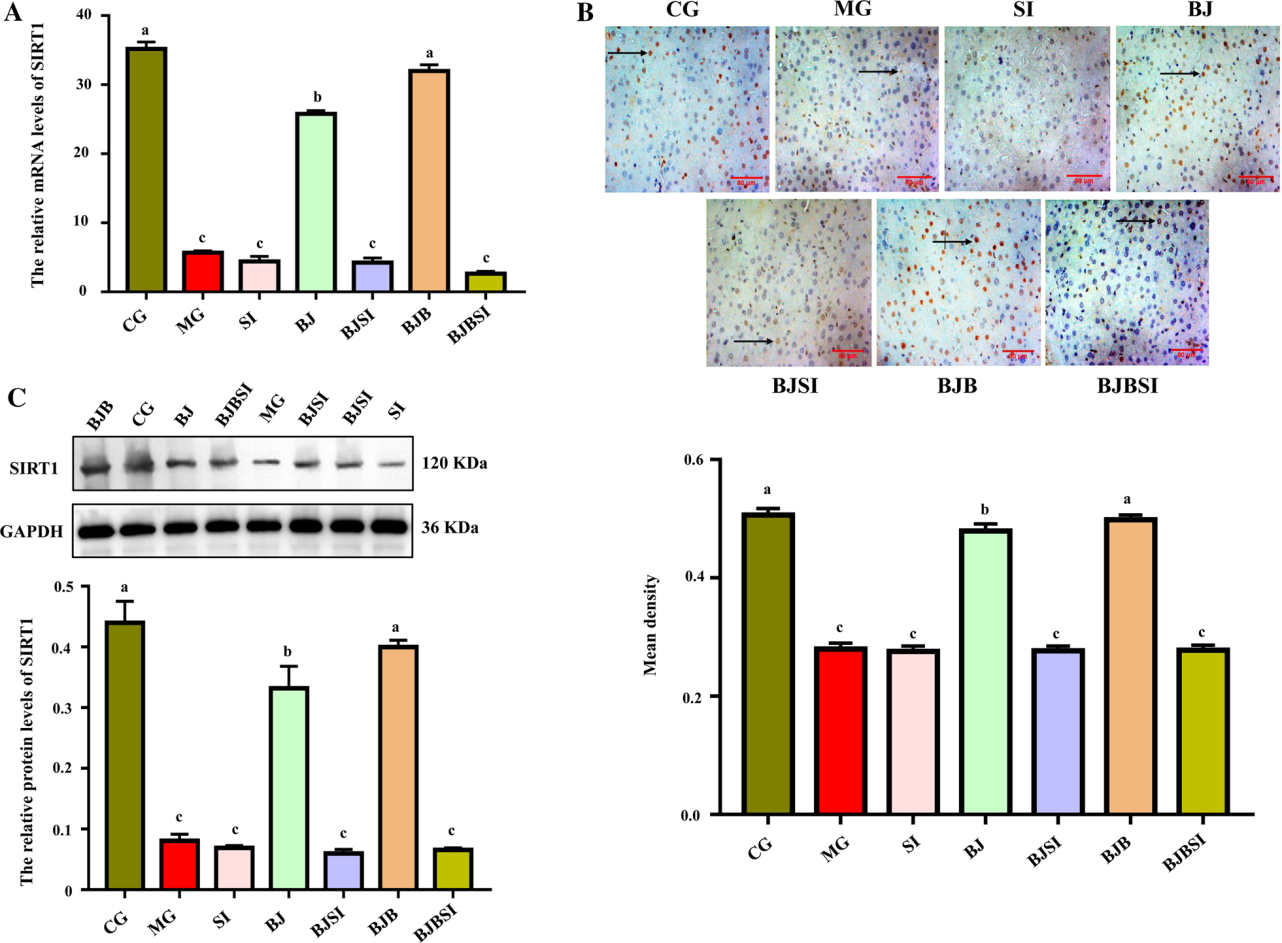
BP restored the decreased level of SIRT1 in AFLD mice. **A** Expression of SIRT1 mRNA in mouse livers was detected by RT-qPCR. Data are mean ± SD. **B** Immunohistochemical analysis was performed to evaluate the expression of SIRT1 in mouse livers of different groups (Scale bar: 80 μm). **C** SIRT1 expression was measured by western blotting. Data are the result of triplicate experiments. Lowercase letters (a, b, c, and d) represent significant differences (*P < 0.05, Student’s t-test, n = 8)

Our earlier study indicated that blueberry or BP restore the mitochondrial structure and function in AFLD mice and that the effect of BP is more potent than that of blueberry alone, while the knockdown of SIRT1 weakens this effect. These findings suggested SIRT1 is essential for mitochondrial protection by BP. RT-qPCR, western blot, and immunohistochemistry (Fig. 4A–C) revealed significant reductions in mRNA and protein levels of SIRT1 in AFLD mice versus normal mice. Interestingly, the SIRT1 expression was elevated in AFLD mice treated with BP, and this effect was reversed by siRNA silencing.

#### BP increased PGC-1α expression via SIRT1

**Fig. 5:**
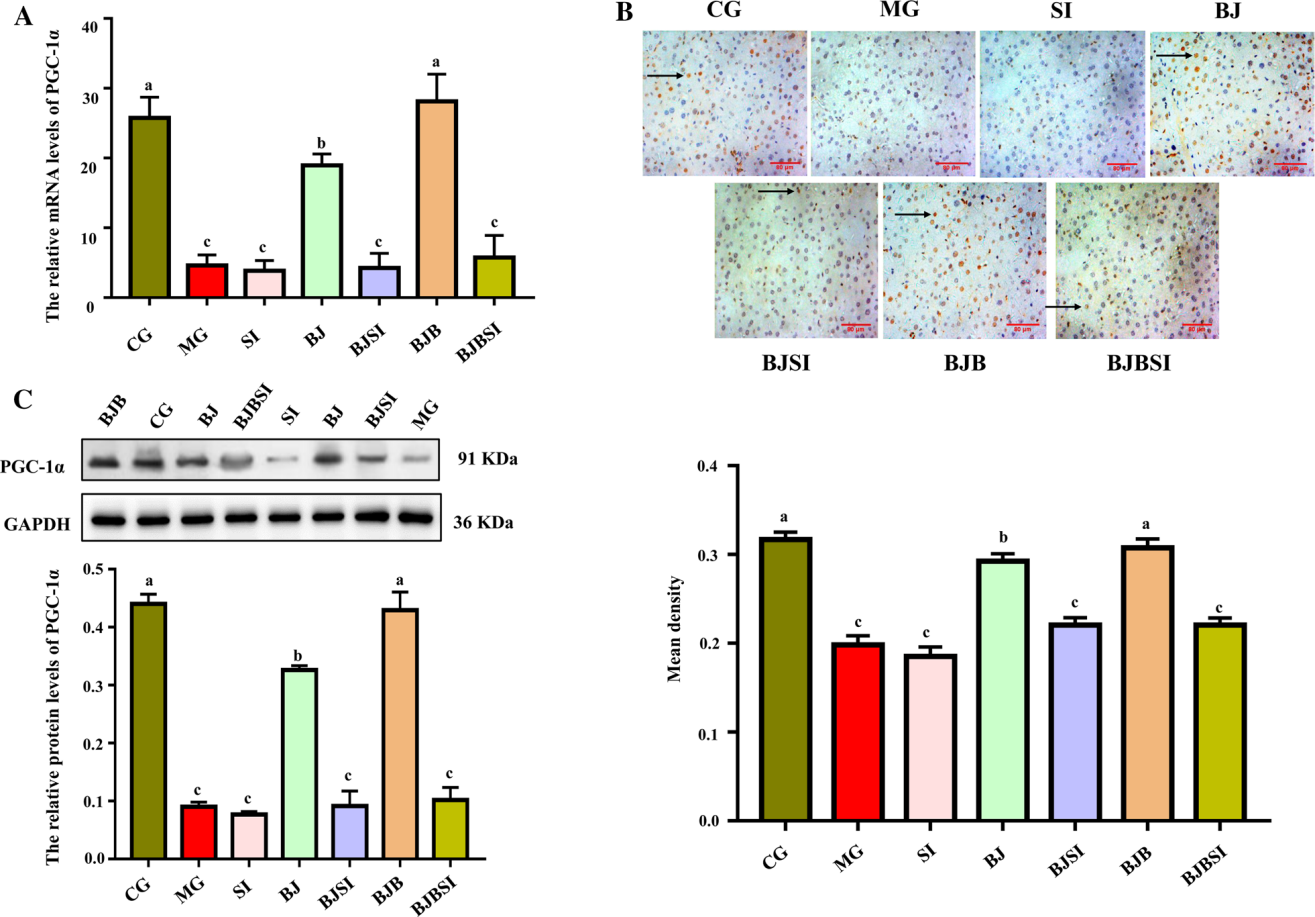
BP increased PGC-1α expression via SIRT1. **A** RT-qPCR of PGC-1α mRNA in mouse liver. PGC-1α expression is expressed relative to GAPDH. **B** Immunohistochemical staining was used to evaluate SIRT1 expression (Scale bar: 80 μm). **C** Western blot analysis of hepatic PGC-1α. Date are shown as mean ± SD, n = 8. Lowercase letters (a, b, c, and d) represent significant differences (*P < 0.05, Student’s t-test, n = 8)

We next sought to understand why SIRT1 knockdown eliminates BP protection of mitochondrial function in AFLD mice. Previous studies have shown that PGC-1α cytokine lies downstream of SIRT1, which is mainly expressed in mitochondria-rich tissues [30]. As a substrate for SIRT1, PGC-1α synthesis and transcriptional activity are catalytically regulated by SIRT1 deacetylase activity [31, 32]. We detected PGC-1α expression (Fig. 5A–C) and observed a significantly decrease in AFLD mice compared to normal mice. After BP treatment in AFLD mice, PGC-1α expression was recovered to normal levels. The impact on PGC1-α by BP was attenuated by SIRT1 silencing.

## Discussion

AFLD represents the most common early stage of alcoholic liver injury, resulting in lipid deposition in hepatocytes, inflammatory cell infiltration, and hepatocytes injury that can be reversed with appropriate intervention and treatment. Left untreated, AFLD will progress to hepatocytes necrosis and irregular hepatocyte regeneration, eventually leading to cirrhosis, for which there is no effective treatment [33]. This study demonstrated that juice restored hepatocyte and mitochondrial structure, respiratory function, and energy metabolism. Mitochondrial oxidative stress was also reduced, suggesting the considerable potential of BP in the treatment of AFLD. The effects of BP were attenuated by SIRT1 silencing and SIRT1 expression was restored by BP, indicating that BP regulates the expression of PGC-1α through SIRT1.

Blueberries contain an abundance of anthocyanins, polyphenols, and flavonoids, which is rare in fruits, and is known as the premier antioxidant of fruits and vegetables [34]. As a micro-ecological regulator of the intestinal tract, probiotics ameliorate endotoxemia and intestinal barrier function and exhibit therapeutic efficacy in AFLD [35]. Blueberries promote the growth of probiotic bacteria, which in turn enhance the biological activity of blueberries, suggesting a synergistic effect [36]. Increasing evidence has shown that blueberries and probiotics may protect the liver from diseases caused by high-fat diets [37], liver fibrosis [38], and hepatocellular carcinoma [39, 40]. BP attenuates the severity of colorectal inflammation and liver injuries caused by exposure to dextran sulfate sodium [41] and mitigates nonalcoholic fatty liver disease via JAK1/STAT3/BAX signaling [42]. Here, using mitochondria as an entry point, we found that BP improved the structure and function of liver mitochondria in an AFLD model, reducing mitochondrial oxidative stress by inhibiting MDA and ROS and activating GSH and SOD. Thus, BP substantially reversed the pathogenesis of alcohol-induced AFLD by regulating mitochondrial function.

A member of the sirtuins, SIRT1 regulates mitochondrial functions, triggering mitochondrial biogenesis by regulating transcription of nucleus-encoded mitochondrial genes [43]. SIRT1 expression increases under hypoxic conditions and is accompanied by mitochondrial elongation, suggesting that SIRT1 regulates this morphological change [44]. SIRT1 overexpression also improves insulin resistance in part by targeting mitochondria [45]. SIRT1 regulates mitochondrial function in embryonic stem cells exposed to oxidative stress [46]. Oxidative stress and mitochondrial dynamics may be associated with SIRT1 activation in muscle cells, limiting oxidative stress [47]. SIRT1 protects against acetaminophen hepatotoxicity by limiting the inflammatory responses and oxidative stress [48]. Similarly, we found that SIRT1 mRNA and protein levels were significantly lower in the AFLD liver than in normal mice and SIRT1 deficiency significantly limited the efficacy of BP in mitochondrial protection and anti-oxidative stress, suggesting that BP attenuates mitochondrial function and oxidative stress through SIRT1.

The transcriptional coactivator PGC-1α is a deacetylated substrate for SIRT1 and plays a critical role in mitochondrial biosynthesis and maintenance [49, 50]. The PGC-1α protein, which is abundant in mitochondria-rich tissues, consists of 795 amino acids with a molecular weight of approximately 92 kDa [51]. PGC-1 is involved in modifying multiple molecular pathways and maintains the stability of mitochondrial function and energy metabolism under a variety of conditions. Activating SIRT1-PGC-1α-mitochondrial biosynthesis can limit alcohol-induced liver injury [52], and the degradation of PGC-1α accounts for hepatosteatosis induced by chronic alcohol overconsumption [53]. PGC-1αexpression significantly increased after BP intervention, while SIRT1silencing inhibited PGC-1α expression and abrogated the effect of BP, indicating that SIRT1 activation of PGC-1α was necessary for BP regulation of hepatocyte mitochondria in AFLD.

## Conclusions

BP synergistically inhibit AFLD by protecting the mitochondrial structure, improving mitochondrial function, and reducing oxidative stress. BP upregulated SIRT1 and PGC-1α, thereby protecting hepatocyte mitochondria, maintaining cellular energy metabolism, and protecting against oxidative damage, reversing liver injury caused by alcohol. Our results suggest BP may hold promise as a dietary supplement to prevent AFLD. Other isoforms of SIRT have not been analyzed in this study, which is a limitation in this study, and will be the subject of future investigation.

## Supplementary Information

**Additional file 1.** Figure S1. Expression of AMPK and p-AMPK

## Data Availability

The datasets used and/or analyzed during the current study are available from the corresponding author on reasonable request.

## Abbreviations

AFLD: Alcoholic fatty liver disease
BP: Blueberry juice and probiotics
ADP/O: ADP/oxidation ratio
RCR: Respiratory control rate
SIRT1: Sirtuin-1
ROS: Reactive oxygen species
RT-qPCR: Reverse transcription-quantitative polymerase chain reaction
SDH: Succinic dehydrogenase
CCO: Cytochrome c oxidase
EC: Energy charge
MDA: Malondialdehyde
SOD: Superoxide dismutase
GSH: Glutathione
PGC-1α: Proliferator-activated receptor-gamma coactivator-1α
SD: Standard deviation
