# Supplementary figures and images for: Combined intake of blueberry juice and probiotics ameliorate mitochondrial dysfunction by activating SIRT1 in alcoholic fatty liver disease
Source: Nutr Metab (Lond). 2021 May 10;18:50. doi: 10.1186/s12986-021-00554-3 (PMC8108333; doi:10.1186/s12986-021-00554-3)

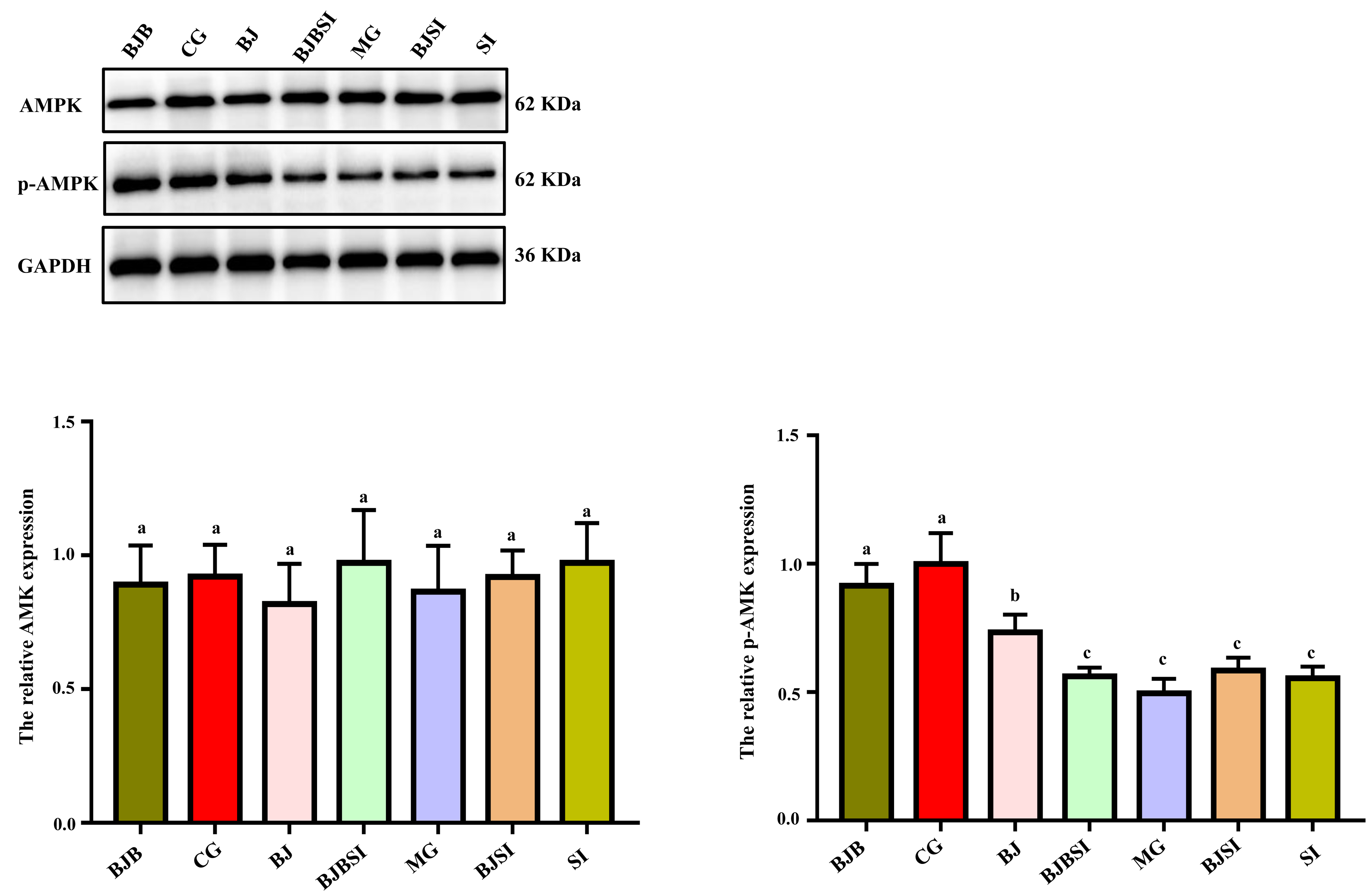

Supplement: Supplementary file 1 — Additional file 1. Figure S1. Expression of AMPK and p-AMPK [file 12986_2021_554_MOESM1_ESM.tif]
